# Determinants and time to blood transfusion among thermal burn patients admitted to Mulago Hospital

**DOI:** 10.1186/s13104-017-2580-2

**Published:** 2017-07-06

**Authors:** C. Kilyewala, R. Alenyo, R. Ssentongo

**Affiliations:** 1 Department of Surgery, Mulago National Referral Hospital, P.O.BOX 7051, Kampala, Uganda; 2 Department of Surgery, Makerere University College of Health Sciences, P.O.BOX 7072, Kampala, Uganda; 3 Plastics and Burns Unit, Department of Surgery, Mulago National Referral Hospital, P.O.BOX 7051, Kampala, Uganda

**Keywords:** Thermal burns, Blood transfusion, Time to transfusion, Determinants of transfusion

## Abstract

**Background:**

Blood transfusion, a practice under re-evaluation in general, remains common among thermal burn patients due to the hematological alterations associated with burns that manifest as anemia. Today advocacy is for restrictive blood transfusion taking into account individual patient characteristics. We went out to identify the parameters that may determine transfusion requirement and the time to blood transfusion for thermal burn patients in Mulago Hospital in order to build statistics and a basis to standardize future practice and Hospital protocol.

**Methods:**

112 patients with thermal burns were enrolled into a prospective cohort study conducted in the Surgical Unit of the Accidents and Emergency Department and Burns Unit of Mulago Hospital. Relevant data on pre-injury, injury and post-injury factors was collected including relevant laboratory investigations and treatment modalities like surgical intervention. Patients were clinically followed up for a maximum period of 28 days and we identified those that were transfused.

**Results:**

22.3% of patients were transfused. The median time to transfusion was 17 days from time of injury and varied with different patient characteristics. The median pre-transfusion hemoglobin (Hb) level was 8.2 g/dL. Transfusion was significantly related to; admission to the intensive care unit (p = 0.001), a body mass index (BMI) <2 kg/m^2^ (p = 0.021), % total burn surface area (TBSA) >20 (p = 0.049), pre-existing illness (p = 0.046), and white blood cell (WBC) count <4000 or >12,000/μL (p = 0.05).

**Conclusion:**

Pre-existing illnesses, a low BMI, TBSA of >20%, admission to the intensive care unit and abnormalities in the WBC count are useful predictors of blood transfusion among thermal burns patients admitted to Mulago Hospital. The precise time to transfusion from time of burns injury cannot be generalized. With close monitoring of each individual patient lies the appropriateness and timeliness of their management.

## Background

Due to the demonstrable complications of transfusion among burns patients in concert with the improved patient outcome on restrictive blood use trials, the role of blood transfusion in burns has undergone re-evaluation over years [1, 2]. These data, have led to recommendations that blood transfusions be used only when there is an apparent physiologic need [3] based on the patients vital signs, estimation of blood loss and evaluation of blood volume, clinical and laboratory evaluation of end organ perfusion.

Current transfusion protocols use a specific (trigger) level of haemoglobin (Hb) or haematocrit (HCT) that dictates when to transfuse packed red blood cells (PRBCs). An acceptable strategy for transfusion of burn patients has not been specifically identified [4]. There is no one ‘common trigger’ but with on-going research, different triggers are adopted. This has changed from the traditional Hb <10 g/dL in the 1960s to <7 g/dL in the 1990s to a range of haemoglobin level of 6–8 g/dL [5–7], which is not used independently but alongside other physiological parameters to decide the need to or not to transfuse the patient.

PRBs transfusion policies at several U.S. burn units surveyed, found that haemoglobin as low as 6 g/dL and haematocrit as low as 15% were acceptable for healthy patients needing limited surgery. The highest haemoglobin considered as a transfusion trigger for critically ill patients with extensive burns and/or burns with cardiopulmonary compromise was 10 g/dL. For the stable burn patients’ recommendations to transfuse were at haemoglobin of ≤8 g/dL for non-critically ill and without cardiopulmonary compromise and for critically ill patients and/or those with cardiopulmonary compromise, transfuse at haemoglobin of ≤10 g/dL [5].

Kwan et al’s study implanting a restrictive transfusion policy in burned children showed that a haemoglobin threshold of 7 g/dL had no more adverse outcomes versus a traditional transfusion trigger of 10 g/dL. In addition, costs incurred to the institution were significantly less [4]. Similar results from other studies are shown [6, 7].

Other strategies to minimize transfusion requirement included minimizing volume of blood lost during surgeries by early wound excision within the first 7 days, the use of the modified tumescent surgical technique using subcutaneous adrenaline and adrenaline saline soaked non adherent pads to reduce intra-operative and total blood transfusion requirement as well as continuous tourniquet application during tangential excision on extremities in burn patients [8–11].

Most studies have defined the transfusion trends in burn patients, focusing on the relationship between % TBSA burn and transfusion needs [12–14].

The rate of transfusion increases with increasing % TBSA burn but with variations for the same burn extents in different study populations e.g. Graves et al. patients with >10% TBSA burn received an average of 19.7 units of blood [13],while for Vasko et al., their patients with >10% TBSA burn required an average of 8.94 units per patient [14]. However this conclusion overly generalizes patients as >10% could mean 11, 25, 70%. For patients with >30% TBSA, the mean transfusion requirement was 17 units [14]. Palmieri et al. found that, on average, 13.7 ± 1.1 units of PRBCs were transfused per patient with >20% TBSA, for burns of >50% TBSA, 30 units of blood were transfused per patient [12]. Approximately 5.7% of patients with <10% TBSA burn, 21% with 11–20% TBSA burn, 39% with 21–30% TBSA burn, and 62% of patients with >30% TBSA burn required a transfusion [15]. This may be attributed to increased surgical blood loss from extensive excision and grafting and also from worsened illness severity leading to impaired erythropoiesis.

Most frequently, on-going blood loss (22%), anaemia (20%), hypoxia (13%) and cardiac disease (12%) are the reasons for transfusion in thermal burn patients, with inhalation burns influencing the decision to transfuse blood in 34% [16]. Acute respiratory distress syndrome (ARDS), and age in a survey of trends of blood transfusion among surgeons were added as other factors increasing transfusion rates apart from the above mentioned [16]. Other reasons for blood transfusion in thermal burn patients were, critical illness extent, other trauma, age, sepsis, need for grafting and, TBSA [16].

Mann et al. found that there was no association of blood transfusion requirement with patient age, timing of the 1st surgical procedure from the time of injury or the length of patient stay in hospital [6].

Considering the available literature on blood transfusion in thermal burns patients, it is clear that little is known about other patient characteristics predisposing to transfusion in burn patients except/other than low haemoglobin levels and % TBSA of burns. However one does not need to labor to explain that factors like, haemodynamic status, nutrition status, may also influence the decision to transfuse at a higher Hb level. Amidst all this, the National Institute of Health consensus conference concluded that no single measurement can replace good clinical judgment concerning the need for RBCs transfusion and this goes for both indication and timing of transfusion.

Generally, compared to other causes of trauma, patients with burn injury are transfused way later from time of injury. In trauma other than burns, the haemoglobin level obtained shortly after injury may not detect occult bleeding or may be confounded by crystalloid related haemodilution but a level <10 g/dL in the first 30 min of patient injury will correctly identify significant blood loss in 90% of trauma patients hence the early transfusion.

In burns, the drop in hemoglobin level is classified as either acute blood loss, well established as the major cause of anaemia in burn injury occurring within the first 1–2 weeks of burn injury, or anaemia of critical illness which occurs between operative events, during wound healing and throughout the resolution of the acute phase of injury and accounts for up to 70% of cases that are transfused. Each of these two categories differs in aetiology from the other [17].

On average, burns patients are transfused 5.2 ± 0.3 days after admission [12]. This has been interpreted that direct thermal injury and sequestration of red blood cells doesn’t have a profound effect on the overall Hb level, otherwise transfusion would be done much earlier as is the case in other causes of trauma.

In another study, burn patients on average were transfused within 28.8 days from time of injury which is normally after all surgeries and relevant procedures required are done [16] however this contradicts the practice of early wound excision, one of the common surgeries performed in burn patients evidenced to decrease blood loss if performed in the first 24 h of injury [9, 18, 19].

Among critically ill patients including burns patients 50% of transfusions occurred in the first 5 days of ICU stay [20]. In the Anemia and blood transfusion in critically ill patients (ABC) study 70% of all transfused patients were transfused in the first 2 ICU days [21] which is still within the first week of admission.

In Mulago Hospital, many burns patients get transfused during their course of treatment however; there is reported scarcity of blood as a resource in across the country mainly due to low responses by citizens to blood donation. This could cause a delay or failure to transfuse patients who may need it.

The transfusion rate and indications of blood transfusion are not documented. The threshold of transfusion possibly a haemoglobin level of <8 g/dL is a typical consideration in our setting. It also varies from one patient to another when clinical and physiological assessments of these patients are considered by the specialist or attending clinician for that matter, as is the case in other burn centers in different countries. Literature on these predictors for the need to transfuse are not well established to allow one to foresee the need for transfusion right from the point of admission in Mulago hence the need for this study to address this gap in information.

## Methods

We carried out a prospective cohort study for a period of 5 months only in Mulago National Referral. Mulago National Referral hospital being one of two such hospitals in the country with a representative population from across the country some self-referred others transferred in from peripheral and far off hospitals across the country. We had no conflict of interest and research funding was by the corresponding Author. We recruited 112 thermal burn patients admitted to the Surgical Unit of the Accidents and Emergency Department within 72 h from the time of the burn injury. Patients with a history of blood transfusions during the episode of injury but prior to admission were excluded.

All patients received standard burns care as per the Burns Unit of Mulago Hospital protocol which included, among others, resuscitation according to the Acute Trauma and life Support (ATLS) protocol, wound care, Fluid replacement according to the parkland formula, pain management, electrolyte replacement and nutritional rehabilitation, infection control, and counseling. The wound care however, did not involve early wound excision. Each patient was prospectively followed up while on the ward for a period of 28 days or until discharge, death or loss to follow up whichever came first. A pretested interviewer administered questionnaire was used to collect data on demographics, history of injury, co-morbidities (respiratory, cardiac disease, hematological disorders and epilepsy among others) and clinical examination findings on severity of burns and systemic examination from consenting participants.

Results from serial laboratory analyses carried out once a week for CBC, LFTs levels at admission and during ward care to diagnose for anaemia, hypoalbuminaemia, and sepsis (determined clinically and white blood cell count of <4000 or >12,000 cell/uL on laboratory analysis) were documented. During the process of following up study participants, we took note of those patients that got blood transfusion; the time of blood transfusion from time of injury, and their Pre transfusion Hb was recorded. There is no blood transfusion protocol in Mulago and the decision to transfuse was collectively made by a team of attending plastic surgeons, residents and general practitioners during ward rounds.

We also reviewed the patients’ files for any other indications of blood transfusion according to the attending team and also if these patients had had any surgery during the study period. We compared the variables above between the patients that received blood with those among patients that did not receive a unit of blood during the period of study. The cut off hemoglobin levels of 11.9–10, 9.9–8, 6–7.9 g/dL and <5.9 were used to stratify patients on basis of HB levels classified into severity groups. The data was checked for errors and corrected, and it was entered in a software package, EPIDATA and exported to a STATA for analysis. Patient confidentiality was maintained and patients’ details were not included in the data set. The hard copies of the questionnaire were kept under lock and key while the soft copy of the data was password protected. Comparisons of demographics and other baseline characteristics of these thermal burn patients were made between patients who received blood transfusion and those who did not receive blood transfusion.

The primary outcome for this analysis was blood transfusion. The study used survival analysis to estimate the time to blood transfusion among patients with different cut offs of TBSA and Hb levels. Kaplan–Meier survival methods were used and time of start of risk was at the date of injury. Patients who were not transfused and alive were censored at day 28. Patients who died or those discharged or lost to follow-up before day 28 were followed up to the date of death, date of discharge or last date seen at the ward whichever came first. A log rank test was used to test for equality of survival for blood transfusion. In a multivariate Cox Proportional Hazard model, the study attempted to identify which factors were associated with blood transfusion among thermal burn patients. All analysis was carried out using STATA 12.1 and a *p* value of p < 0.05 was considered as of statistical significance.

## Results

A total of 161 patients were admitted with burns injuries at Mulago Hospital. Of these, one hundred and twelve (112) patients were recruited into the study during the study period of 5 months. Patient variables were categorized into pre-injury, injury and post-injury factors as in Table 1.

**Table 1:** Description of study participants

Variable	Frequency	Percentage (%)
*Distribution of burns patients by pre*-*injury factors*		
Sex		
Male	75	67.0
Female	37	33.0
Age group		
<5	66	58.93
5–12	10	8.93
>12	36	32.14
Body mass index		
Above normal	19	16.9
(18.5–25 kg/m^2^) normal	59	52.7
Below normal	34	30.4
Pre-existing illness		
Non	93	83.0
Any blood disorders	2	1.8
Respiratory disease	7	6.3
Epilepsy	6	5.4
Others	4	3.6
*Distribution of burns patients by injury related factors*		
Cause of burn		
Scalds	74	66.1
Flame	37	33.0
Contact	1	0.9
Body part involved		
Face	2	1.8
Head and neck	1	0.9
Trunk	1	0.9
Extremities	5	4.5
Perineum	1	0.9
Multiple parts	102	91.1
Degree/depth of burns		
Partial superficial	99	88.4
Partial deep	4	3.6
3rd degree	2	1.8
Mixed	7	6.3
Inhalation injury		
Present	28	25
Absent	84	75
Severity of the burns		
Minor	1	0.9
Moderate	29	25.9
Major	82	73.2
Percentage of TBSA		
Less than 20	62	55.4
20 and above	50	44.6
WBC count at admission		
Normal	66	58.9
Abnormal	46	41.0
Hb count at admission		
10–11	16	14.3
8–9.9	32	28.6
6–7.9	2	1.8
>11	62	55.4
Other injury		
Yes	4	3.57
No	108	96.43
Total	112	100.00
*Distribution of burns patients by post injury factors*		
Surgical procedures performed		
Yes	13	11.61
No	99	88.39
Disposition/ward		
ICU	26	23.21
Holland	86	76.79
Type of admission		
Primary	92	82.14
Referral	20	17.86
WBC count during admission		
Yes	64	57.14
No	49	42.86
Source of infection		
Yes	49	43.75
No	63	56.25
Low haemoglobin level		
Yes	68	60.71
No	44	39.29
Lowest serum albumin		
Normal (3.5–4.9)	25	22.32
>2.9–3.5	23	20.54
2.5–2.9	15	13.39
Severe (<2.5)	49	43.75

We classified variables as injury, pre injury and post injury factors to single out which factors risk a patient to blood transfusion

A total of 112 patients were recruited. 67% of these were male. A larger percentage was children under 5 years of age and commonly due to lack of adult supervision of these children back at home. 30% of the participants were admitted with an initial below average Basal metabolic rate range whereas 52% were within normal BMI range. Comorbidities including epilepsy were observed in 17% of the participants

The most common cause of these burns was scalds (66.1%). And among patients with open flame burns, 25 presented with inhalation injury

Approximately 12% of the participants had surgery (skin grafting, Escharotomy or amputation) whereas we offered routine wound care to all participants. 99% of the patients were moderately or severely injured and 23.21% ended up in the burns intensive care unit

### Time to blood transfusion

**Fig. 1:**
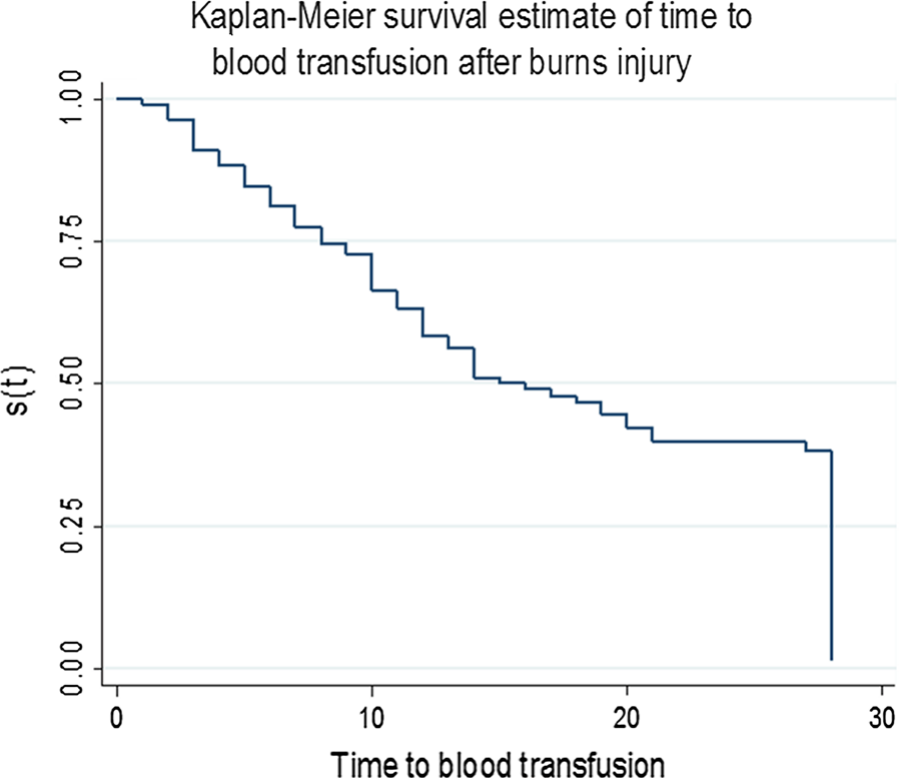
Survival curve for the time to blood transfusion. Study participants that we transfused with blood got the first unit within the first month from time of injury. The median time [(t) = 0.5] of transfusion from this figure is 17 days. Chances of blood transfusion were very high with in the first 3–21 days unlike in the immediate post injury time which can be argued that the fluid of choice in our practice is ringers lactate in the first 24 h and remains crystalloids for maintenance or replacing deficits. Also, that the ostensible Hematocrit/hemoglobin values for an initial haemo-concentration and later dilution in this period are highly influenced by the severity of injury and resuscitation and true physiological values are achieved after about 3 days from time of injury

The median time to blood transfusion was 17 days [s(t) = 0.5]. Chances of blood transfusion among thermal burn patients were very high within 3–21 days of admission. Beyond 21 days the risk of surviving blood transfusion was constant as shown in Kaplan curve in Fig. 1.

This median time to transfusion remained >14 days from time of injury irrespective of where on the burns unit one was admitted. However, patients in the burns ICU where transfused slightly earlier than those admitted to the general ward (Holland).

### Determinants of blood transfusion

Univariate analysis: see Table 2.

**Table 2:** Differentials in time to blood transfusion

Variable	Frequency	Log rank χ^2^	p value
*By pre-injury related factors*			
Sex of the child			
Male	75	2.32	0.1274
Female	37		
Age group			
<5	66	0.18	0.6695
5–12	10		
>12	36		
Body mass index (18.5–25 kg/m^2^)			
Above normal	19	11.68	0.1115
Normal	59		
Below normal	34		
Pre-existing illness			
Non	93	4.16	0.3849
Any blood disorders	2		
Respiratory disease	7		
Epilepsy	6		
Others	4		
*By injury related factors*			
Degree/depth of burns			
Partial superficial	99	1.07	0.7832
Partial deep	4		
3rd degree	2		
Mixed	7		
Inhalation injury			
Absent	28	8.85	0.0029
Present	84		
Percentage of TBSA			
Less than 20	62	11.37	0.0007
20 and above	50		
Cause of burn			
Scalds	74	12.04	0.0024
Flame	37		
Contact	1		
Severity of the burns			
Minor	1	8.01	0.018
Moderate	29		
Major	82		
Baseline WBC count at admission			
Normal	63	1.13	0.2874
Abnormal	48		
Baseline Hb count admission			
11–10	16	1.78	0.6189
8–9.9	32		
6.7.9	2		
>11	62		
*By post injury factors*			
Surgical procedures performed			
Yes	13	6.56	0.0104
No	99		
Ward			
ICU	26	22.58	0.0000
Holland	86		
Abnormal WBC count during admission			
Yes	63	1.86	0.1729
No	48		
Focus of infection			
Yes	49	3.02	0.0821
No	63		
Lowest haemoglobin level			
Yes	62	5.11	0.0238
No	43		
Lowest serum			
Normal (3.5–4.9)	24	12.92	0.0048
>2.9–3.5	23		
2.5–2.9	15		

Univariate analysis for influence on tie to transfusion. Categorized as pre-injury, injury and post-injury factors in this table, we found that

Sex, body mass index (BMI) and pre-existing illnesses had significant association with blood transfusion. However, there was no association between patient age group and blood transfusion

We observed a significant association of blood transfusion with inhalation injury, percentage of TBSA, cause of burn and baseline WBC count. However, there was no association with baseline HB level, body part injures or depth of the burns

All the post injury variables had significant association with time to blood transfusion (surgical procedures performed, ward unit on which patient is admitted, WBC count during admission, focus of infection, low haemoglobin level during admission, lowest Serum albumin during admission) (p < 0.05) except for weather the patient was a primary or secondary referral to Mulago

#### Pre injury factors

Sex, body mass index (BMI) and pre-existing illnesses had significant association with blood transfusion. There is no association between patient age group and blood transfusion.

#### Injury related factors

We observed a significant association of blood transfusion with inhalation injury, percentage of TBSA, cause of burn and baseline WBC count. However, there was no association with baseline HB level, body part injures or depth of the burns.

#### Post injury factors

Assessing for association of blood transfusion with post injury factors, all the variables had an association with time to blood transfusion (surgical procedures performed, ward unit on which patient is admitted, WBC count during admission, focus of infection, low haemoglobin level during admission, lowest Serum albumin during admission) (p 0.05) except for weather the patient was referred to Mulago from another health center or not.

### Determinants of blood transfusion

#### Bivariate analysis

A bivariate analysis of categorical variable was done to ascertain hazard ratios and statistical significance of the different variables with blood transfusion.

Similar statistical findings in this model as with the log rank test were seen with statistical significant association (p < 0.05) for a low BMI, presence of inhalation injury, >20% TBSA, scald injury, major burns, patient being admitted in the ICU, undergoing surgery, experiencing a low HB count, having a focus of infection, and a deranged WBC count during admission.

No statistically significant association was found between transfusion and patient age groups, baseline laboratory findings for haemoglobin, and white blood cell counts.

#### Multivariate analysis

At this point, variables whose p values were greater than 0.2 from the bivariate analysis were dropped and not used for further analysis to maintain an acceptable number of categories. Whereas the hazard ratios varied for subcategories of the different variables, only % TBSA greater than 20%, BMI below normal, pre-existing illnesses, abnormal white cell counts during admission and admission to the ICU showed statistical significance (p < 0.05). The risk of transfusion when admitted to Holland was 0.153 times less than that if admitted to the ICU (95% CI 0.051–0.458). This was statistically significant (p = 0.001).

Among patients with a less-than-normal BMI, the risk of blood transfusion was 3.724 times that of patients with a normal BMI (HR 3.26; 95% CI 1.26–8.23) and this finding was statistically significant (p = 0.13). The absence of a preexisting illness lowered the risk for blood transfusion by approximately 10% (HR 0.103; 95% CI 0.01–0.959, P = 0.046) as seen in Table 3.

**Table 3:** Determinants of blood transfusion

Variable	HR	p value	95% confidence interval
*Multivariate analysis*			
Cause of burn			
Scalds	1.00	–	–
Flame	0.880	0.850	0.234–3.304
Inhalation injury			
Absent	1.00	–	–
Present	2.430	0.251	0.534–11.068
Sex			
Male	1.00	–	–
Female	1.323	0.571	0.503–3.477
Percentage of body burnt			
<20	1.00	–	–
>20	3.340	0.049	1.005–11.102
Pre-existing illness			
Yes	1.00	–	–
No	0.103	0.046	0.011–0.959
Body mass index			
(18.5–25 kg/m^2^) normal	1.00	–	–
Above normal	1.211	0.765	0.344–4.268
Below normal	3.724	0.021	1.220–11.367
Ward			
ICU	1.00	–	–
Holland	0.153	0.001	0.051–0.458
Low haemoglobin			
Yes	1.00	–	–
No	0.695	0.644	0.149–3.240
Source of infection			
Yes	1.00	–	–
No	0.808	0.746	0.223–2.928
Surgical procedures			
Yes	1.00	–	–
No	0.509	0.246	0.163–1.594
Deranged WBC during admission			
Yes	1.00	–	–
No	0.295	0.057	0.084–1.035

From this table, the likelihood of transfusion were compared for different variables and expressed as hazard ratios. This was higher for patients who suffered inhalation injury, TBSA > 20%, low BMI (<18.5 kg/m^2^), patients admitted to the ICU, low Hemoglobin level, patients with features of infection and those that underwent surgery

Despite the Hazard ratios, only patients with; percentage TBSA injured >20%, BMI below normal, pre-existing illnesses, abnormal white cell counts during admission and admission to the ICU showed statistical significance (p < 0.05)

Patients whose TBSA > 20% had a 3.34 times risk of being transfused than the patients whose TBSA is less than 20% (95% CI 1.005–11.102) with a p value of 0.049.

The risk of blood transfusion with a normal range of white blood cell count (between 4000 and 12,000) was reduced by approximately 30% compare to patients with an altered WBC count (HR 0.295; 95% CI 0.084–1.035 p = 0.057).

There was not statistical significance in the risk of transfusion between male and female patients, different causes of thermal burns, and presence of inhalation injury, a low level HB recording, and presence of a focus of infection or patient having undergone a surgical procedure. The p values were 0.571, 0.850, 0.251, 0.646, 0.746 and 0.246 respectively.

## Discussion

### Prevalence of blood transfusion

Blood transfusion is the most effective way to treat anemia of thermal injury and hence is common practice among thermal burn patients. It is also indicated for pre-existing illnesses like cardiac disease and, hypoxia among other conditions in this category of patients. And even a more common practice if the patient has undergone an operation during the period of management in the developed countries. However, due to the known complications with this practice, trends to restrict blood transfusion have been encouraged.

In this study, 22.3% of patients were transfused with blood. This was a higher finding compared to the 11% prevalence by Yogore et al. in 2006 among thermal burns patients [15]. Yasser et al. in German in 2010 following a retrospective study on adult (>18 year old) surgical ICU patients reported that 30.9% were transfused [22]. This is still a lower prevalence compared to a 46.2% incidence of transfusion among burn ICU patients in this study.

It is difficult to analyse why the prevalence was higher in this study with, however it is worth noting we didn’t have a standard care for blood transfusion against which this was assessed in our unit and 68% of the decision to transfuse was based on clinical judgment.

### Time to blood transfusion

The median time to transfusion in this study was 17 days from time of thermal burns injury. This correlates with findings by Palmieri et al. in 2004 who, after a survey, reported that burns patients were transfused within the first 28.8 days post injury [16]. However, the same author reports, in 2006 an average time to transfusion among burns patients of 5.2 ± 0.3 days. Early transfusion rates are associated with surgery which could in fact be the practice of early wound excision, a common practice in the management of burns in different facilities. Unfortunately this is not a practice in Mulago Hospital hence the later time to blood transfusion.

Napolitano et al. in 2009 similarly found that patients in the ICU including burn patients were transfused in the 1st 5 days [20]. ICU and critical care studies also report early transfusion in the 1st 1 week of admission to the ICU [21].

The indications for transfusion in this study were 16% based on laboratory findings, 68% on clinical judgment and 16% on both.

With these variations in time to blood transfusion across studies, it is ostensibly arguable that emphasis on personalizing blood transfusion to each individual patient, their clinical and physiological status, as was the case in this study rather than waiting for arbitrary trigger Hb levels is important, paradigms shift. But also to note is that earlier transfusion has shown better outcome in patients compared to late transfusion [22], therefore one needs to be keen from the start. This is where using key determining factors comes in handy.

### Determinants of blood transfusion

This study set out to identify the determinants of blood transfusion among thermal burn patients during their stay on the burns for 28 days. These factors where categorized as pre-injury, injury and post-injury factors in this study.

Among the pre injury factors, preexisting illness was shown to be the only pre injury variable with statistical significance with blood transfusion. In this category were illnesses like respiratory diseases, cardiac disease, and epilepsy, HIV, among others. The risk for blood transfusion was increased by approximately 90%. This is in line with a number of studies that quote preexisting illness as a determinant of transfusion mainly including cardiorespiratory illnesses, and preexisting anemia [15, 16, 20].

No association was reported in previous studies to exist between blood transfusion and BMI as was found in this study with a significant p value of 0.001 for patients that had a lower than 18.5 kg/m^2^ BMI. It would have been of more value if further description of whether or not these patients were malnourished however, we didn’t include all parameters than define malnutrition in burn patients. Reports on poor nutrition in burns with consequential drops in serum iron and vitamin B 12 levels may explain a nutritional anemia.

Age below 5 even as it was associated with blood transfusion, it didn’t show statistical significance as a determinant of blood transfusion in this study compared to other studies [6, 16, 21].

Injury related factors included; cause of thermal burns (scalds, flame or contact), extent of injury in terms of % TBSA, depth severity and body part involved, presence of inhalation injury and any other injuries incurred at time of thermal burns.

Of these, statistical significance was found with a percentage TBSA greater than 20%, presence of inhalation injury, as in a survey by Palmieri et al. [16] and studies [12, 13, 15, 20]. Admission to the ICU, which correlates with severity of burns injury or how critically ill a patient was found to be, was also seen to be a significant determinant. In terms of severity of illness, Posluszny et al. in 2010 used APACHE scores and duration of critical illness [17], that showed correlation of critical illness and blood transfusion. A patient was at a higher risk of transfusion if critically ill and was a significant determinant of blood transfusion. This is in agreement with this study.

Among the post injury factors, abnormally low or high white blood cell count during admission of <4000 or >12,000 cells/μL was captured in as one of the haematological alterations. It could point to infection in burn patients, among other causes. Whereas studies have investigated these alterations, no association was reported with blood transfusion in previous studies as was found statistically significant in this study (HR 0.295 for patients with a normal WBC count; 95% CI 0.084–1.035, p = 0.05).

In line with the continuum of sepsis, this study found that the risk of transfusion given that a patient had no focus of infection was decreased by up to 0.4. And 0.5 times if they maintained a normal white cell count between 4 and 12 thousand cell/μL. Studies have shown that infection per se is useful in determining risk of blood transfusion [16] and this is not far from the interpretation of the findings above.

Whereas a haemoglobin level of greater than 11 g/dL decreased the risk of blood transfusion by approximately 70% and increased by approximately 3 times for HB levels less than 10 g/dL. This finding is in line with recommended strategies against liberal blood transfusion at HB levels higher than 10 g/dL but rather the restrictive blood transfusion strategies which is well tolerated even at the level of critical care in ICU [1, 4, 23–26].

## Conclusion

- The prevalence of blood transfusion in thermal burn patients is 22.3% in Mulago Hospital is slightly higher compared to that found in other studies.
- The median pre transfusion HB level of 8.2 g/dL is within the recommended range.
- The median time to blood transfusion from time of injury in these patients is 17 days but this cannot be generalized but rather a patient specific measure.
- Pre injury factors that are associated with blood transfusion include; female gender, preexisting illnesses and a lower than normal BMI. The injury related factors are; scalds injury, TBSA greater than 20%, presence of inhalation injury and the post-injury factors are patients admitted to the ICU, a low haemoglobin level and a deranged WBC count during admission, having a focus of infection, undergoing a surgical procedure.
- The presence of pre-existing illnesses, a low BMI, TBSA of >20%, admission to the ICU and abnormalities in the WBC count are useful determinants of blood transfusion among thermal burns admitted to Mulago Hospital.
- We could, in future assess the effect of blood transfusion on burns patients in our setting to justify the need to limit transfusion rates.

## Limitations

- The study was carried out in the burns unit of a tertiary hospital which may have a distinct set of patients that is from many other facilities. The care and infrastructure may not be used to generalize standards for burn care.
- Lack of a standard transfusion protocol for Mulago Hospital burns unit hence lack of standard criteria for transfusion.
- Varying number of attending surgeons and health workers including transitioning residents and intern doctors may influence the varied indications of transfusion at different points in time that may not have been captured to explain the differences in decision making to transfuse or not transfuse thermal burns patients especially across the same level of hemoglobin.

## Abbreviations

Hb: haemoglobin
ICU: intensive care unit
HCT: haematocrit
CBC: complete blood count
TBSA: total body surface area
RBC: red blood cell
PRBCs: packed red blood cells
PCV: packed cell volume
LFT: liver function tests
BMI: body mass index
WBC: white blood cell
